# Curative resection after percutaneous drainage followed by preoperative panitumumab monotherapy for locally advanced sigmoid colon cancer with intra-abdominal abscess: a case report

**DOI:** 10.1186/s40792-023-01800-x

**Published:** 2024-01-18

**Authors:** Yusuke Asada, Fumitaka Nakayama, Takashi Takenoya, Ippei Oto, Tetsuya Nakamura, Koji Osumi, Noriaki Kameyama

**Affiliations:** Department of Surgery, Ogikubo Hospital, 3-1-24 Imagawa, Suginami, Tokyo 167-0035 Japan

**Keywords:** Locally advanced colon cancer, Neoadjuvant chemotherapy, Panitumumab, Intra-abdominal abscess, Percutaneous drainage

## Abstract

**Background:**

The gold standard treatment for locally advanced colon cancer is curative surgery followed by adjuvant chemotherapy, although this approach is associated with serious concerns, such as high recurrence rates and occasionally unnecessary oversurgery. Neoadjuvant chemotherapy may be a promising strategy for overcoming these issues. This study reports a case of a recurrence-free patient who underwent curative resection without significant organ dysfunction after preoperative chemotherapy for locally advanced sigmoid colon cancer. The tumor coexisted with a large intra-abdominal abscess, and the patient was quite frail at the first visit. We performed percutaneous drainage followed by preoperative panitumumab monotherapy, which yielded favorable outcomes.

**Case presentation:**

A 78-year-old frail woman was emergently transferred to our hospital with fever and abdominal pain. The diagnosis was locally advanced sigmoid colon cancer stage IIIC (T4bN2aM0) with a large intra-abdominal abscess. Immediate curative surgery was inappropriate, considering both tumor progression and the patient’s frailty. We performed percutaneous drainage and colostomy construction, which was followed by seven cycles of preoperative panitumumab monotherapy without significant adverse events. After these treatments, inflammation was well controlled, and the tumor shrank remarkably. Furthermore, the patient recovered well from frailty; therefore, curative sigmoidectomy combined with resection of the left ovary and stoma closure was possible without any postoperative complications. The final pathological finding was T3N0M0, stage IIA disease. The patient was recurrence-free and had no significant organ dysfunction 21 months after the curative surgery.

**Conclusions:**

The management of intra-abdominal abscesses and tailor-made preoperative chemotherapy based on the patient’s frailty may have been the key factors responsible for the favorable course of this patient. Although further research is needed on the appropriateness of percutaneous drainage for malignancies related to intra-abdominal abscesses and preoperative panitumumab use for locally advanced colon cancer, the study findings can serve as reference for managing similar cases in an aging society.

## Background

The gold standard treatment for locally advanced colon cancer (LACC) without distant metastasis is curative surgery, which includes combined resection of adjacent organs followed by adjuvant chemotherapy (ACT). However, the oncological and functional outcomes of this strategy are not satisfactory. The recurrence rate after this procedure has been reported to be high (15–43%) [1, 2], and this approach may occasionally lead to unnecessary multivisceral loss to maintain an overly safe resection margin, especially if the tumor is complicated by inflammation, which is another serious concern.

In light of these concerns, neoadjuvant chemotherapy (NAC) for LACC has recently become a promising and novel strategy. Here, this study reports a case involving a recurrence-free patient who underwent curative resection without significant organ dysfunction after preoperative chemotherapy for locally advanced sigmoid colon cancer. The tumor coexisted with a huge intra-abdominal abscess, and the patient was 78 years and quite frail at the first visit. Considering the patient’s frailty, we performed percutaneous drainage followed by preoperative panitumumab (Pmab) monotherapy, which resulted in a favorable course.

## Case presentation

**Fig. 1:**
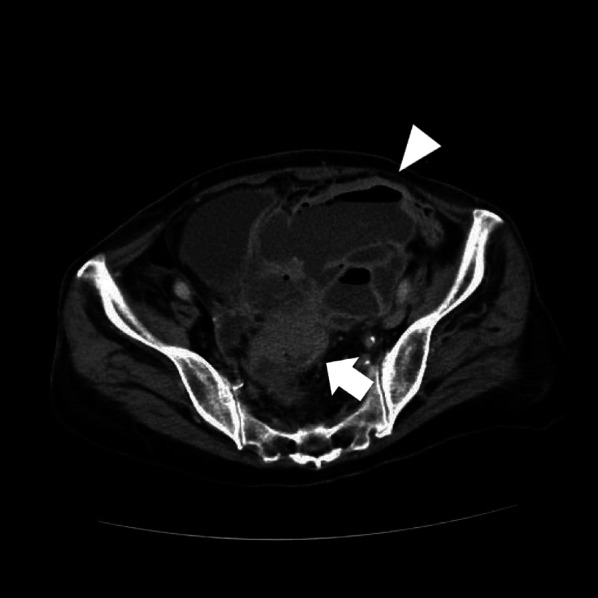
CT examination performed at the first visit. Sigmoid colon cancer (arrow) with a large intra-abdominal abscess (arrowhead) was suspected

A 78-year-old woman was emergently transferred to our hospital with fever and abdominal pain. The patient was generally ill and almost immobile. Her medical history included a total abdominal hysterectomy for uterine fibroids. Her body temperature was 37.6 ℃, white blood cell count was 16,700 cells/µL, and C-reactive protein level was 19.33 mg/dL. Computed tomography (CT) revealed sigmoid colon cancer with a huge intra-abdominal abscess approximately 13 cm in diameter (Fig. 1). No diverticula was observed in the patient. While the exact location of perforation was unclear, the perforation seemed to be at a micro level within the tumor, mainly affecting the mesenteric and/or retroperitoneum side, suggesting that diffuse peritonitis was likely avoided. Therefore, we performed emergent percutaneous drainage in addition to bowel rest and antibiotics. Inflammation was well-controlled within a week. Cytological analysis of the drained fluid revealed inflammation without significant malignancies.

**Fig. 2:**
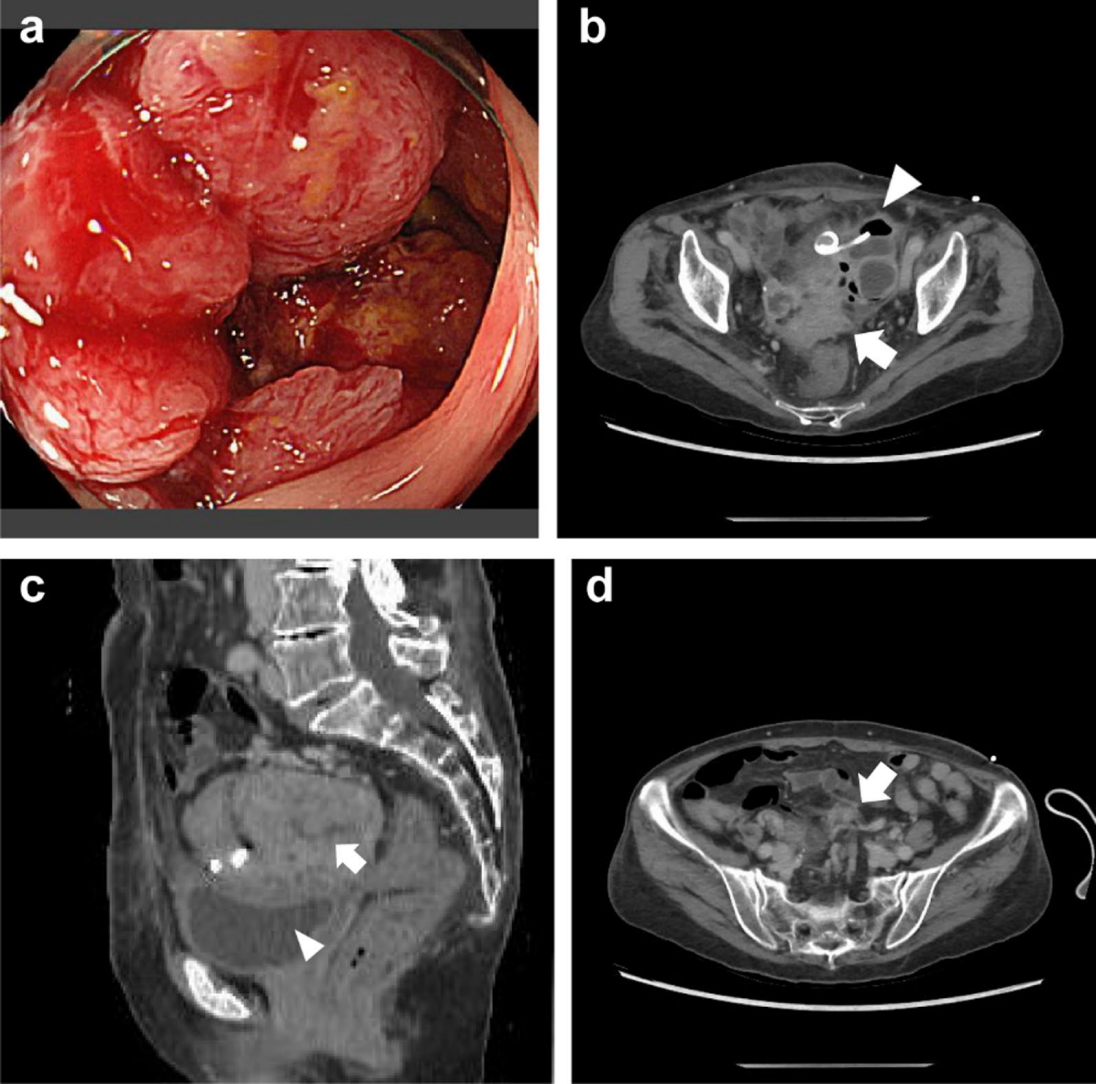
Colonoscopy and re-evaluation CT after percutaneous drainage. Colonoscopy revealed circumferential advanced cancer in the sigmoid colon (**a**). CT revealed a bulky tumor possibly invading the adjacent organs (arrow), and the drainage tube with residual abscess (arrowhead) (**b**). The fat layer between the tumor (arrow) and the bladder (arrowhead) was disappeared (**c**). Enlarged regional lymph nodes were also observed (arrow) (**d**)

Colonoscopy revealed circumferential advanced cancer in the sigmoid colon, and a biopsy revealed tubular adenocarcinoma (Fig. 2a). Tests for both RAS and BRAF showed wild-type status, and those for tumor markers showed negative results. Follow-up CT for re-evaluation revealed a bulky tumor that possibly invaded the adjacent organs (such as the bladder, ileum, and ovary), with enlarged regional lymph nodes (Fig. 2b–d). Although distant metastasis was not suspected, the diagnosis was LACC, stage IIIC (T4bN2aM0), according to the 8th Union for International Cancer Control classification. The tumor was complicated by inflammation, including a residual abscess. The patient also exhibited pleural effusion, primarily due to undernutrition, with a serum albumin level of less than 2 g/dL, lymphocyte count of 835 cells/µL, and total cholesterol level of 160 mg/dL. Immediate curative surgery including multivisceral resection was inappropriate from the viewpoint of both local tumor progression and the patient’s frailty; therefore, we decided to perform preoperative chemotherapy first.

**Fig. 3:**
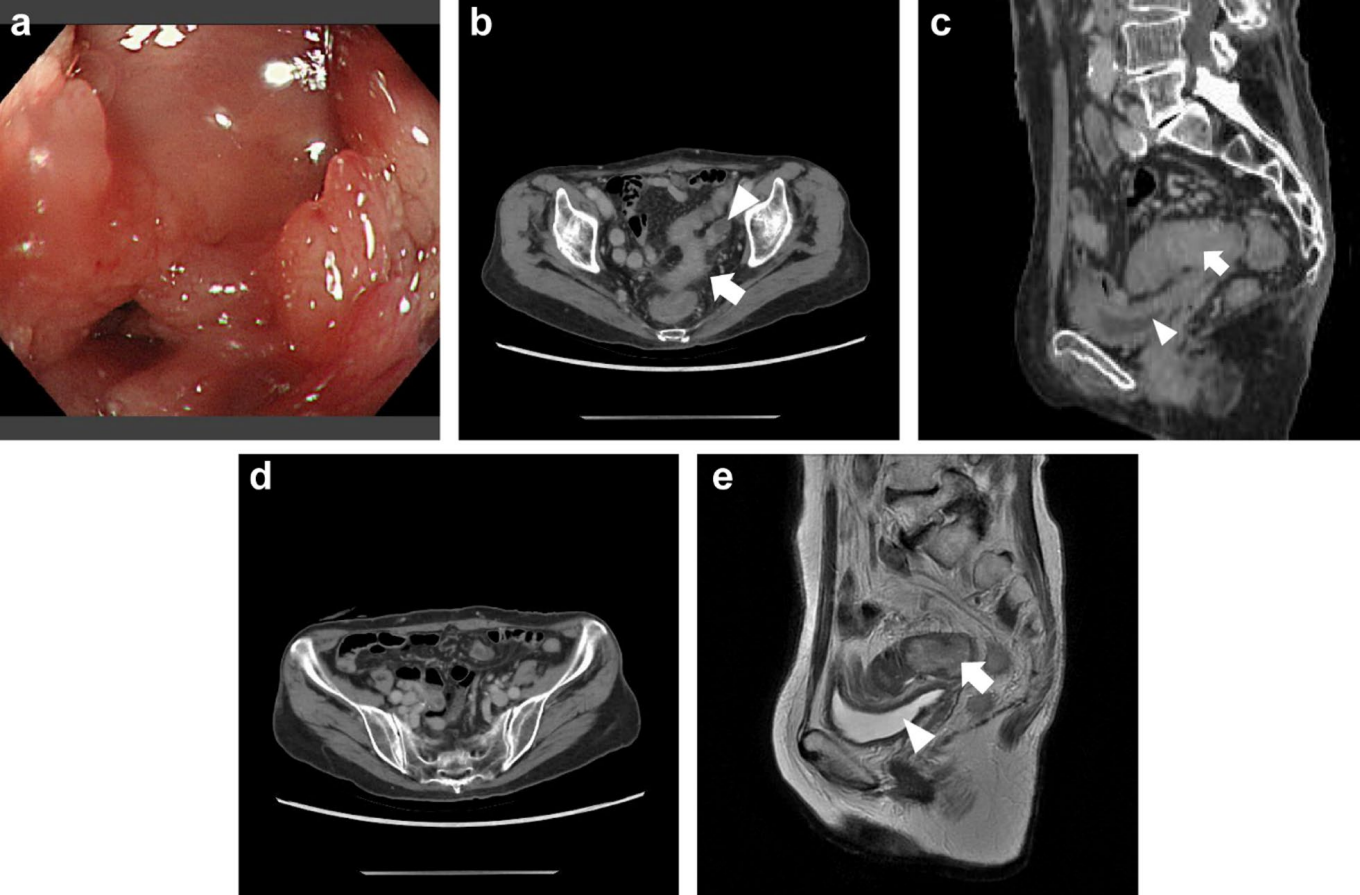
Examinations after preoperative chemotherapy. The tumor shrank with flattening on colonoscopy (**a**). On CT, the tumor also shrank remarkably (arrow), although invasion of the left ovary (arrowhead) was still possible (**b**). The fat layer between the tumor (arrow) and the bladder (arrowhead) was observed (**c**). Enlarged regional lymph nodes were disappeared (same slice level as Fig. 2d (**d**). Magnetic resonance imaging also revealed that invasion of the bladder was negative: the fat layer between the tumor (arrow) and the bladder (arrowhead) was preserved (**e**)

We performed laparoscopic loop colostomy construction in the right transverse colon and initiated chemotherapy. Pmab monotherapy (6 mg/kg intravenously on day 1 every 2 weeks) was selected for this frail patient, and seven cycles were completed without significant adverse events. After chemotherapy, the tumor shrank with flattening on colonoscopy (Fig. 3a). The tumor and enlarged regional lymph nodes also shrank significantly on CT. While the possibility of left ovary involvement still existed, invasion of the bladder and ileum was no longer suspected (Fig. 3b–d). Magnetic resonance imaging and cystoscopy were performed, showing that invasion of the bladder was negative (Fig. 3e). Radiological assessment revealed a partial response, according to the Response Evaluation Criteria in the Solid Tumors guidelines. Notably, the previously observed intra-abdominal abscess and pleural effusion disappeared, and the patient’s frailty, including nutritional status, was improved, with serum albumin level increasing to approximately 3 g/dL, lymphocyte count increasing to 1,750 cells/µL, and total cholesterol level increasing to 284 mg/dL. Therefore, we decided to perform curative surgery.

**Fig. 4:**
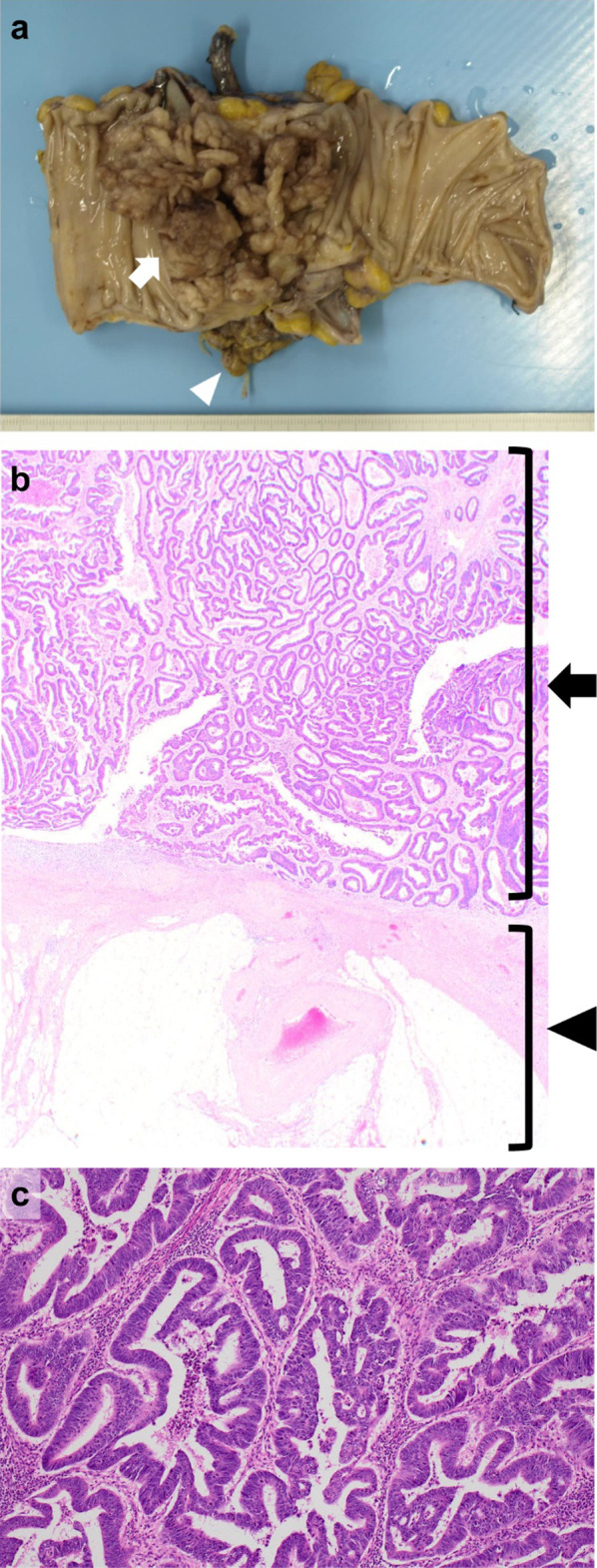
Specimen of curative surgery. Curative sigmoidectomy and combined resection of the left ovary were performed: the tumor (arrow) and the left ovary (arrowhead) (**a**). The final pathological finding was T3: the muscularis propria was disappeared with tumor invasion (arrow), although a normal subserosal layer was preserved outside (arrowhead) (hematoxylin and eosin, × 20) (**b**). The histological type was well and moderately differentiated tubular adenocarcinoma, and despite a significant radiological response the pathological tumor regression was judged to be minimal (hematoxylin and eosin, × 100) (**c**)

Curative open sigmoidectomy with regional lymphadenectomy (high ligation of the inferior mesenteric artery), combined resection of the left ovary, and stoma closure were performed (Fig. 4a). Notably, the abscess had completely disappeared in the surgical finding. The operation time was 264 min, and estimated blood loss was 130 mL, with no need for blood transfusion. The postoperative course was uneventful. The final pathological finding was well and moderately differentiated tubular adenocarcinoma, T3N0M0, stage IIA, and complete resection with a sufficient resection margin had been achieved. Although the radiological response was significant, the pathological assessment indicated only minimal tumor regression (Fig. 4b, c). The patient preferred ACT with oral fluoropyrimidine for 6 months, which was completed without significant adverse events. The patient was recurrence-free and had shown no significant organ dysfunction 21 months after the curative surgery.

## Discussion

This study presents a successful case of curative resection after percutaneous drainage followed by preoperative Pmab monotherapy for locally advanced sigmoid colon cancer with a huge intra-abdominal abscess in an elderly and frail patient. The two important aspects of the management of this case are described in the following paragraphs.

The first important aspect was the management of intra-abdominal abscess at the first visit. Although colorectal cancer with an intra-abdominal abscess has been reported occasionally, its management is complicated. While complete resection, including whole-abscess resection, is the primary principle of colorectal cancer surgery, it may be impossible in some cases. Severe inflammation makes accurate identification of resection lines difficult and weakened abscess walls can rupture easily. Pus in an abscess may contain cancer cells from microperforations of the tumor; therefore, rupture of the abscess and uncontrollable spread of pus into the abdomen can result in recurrence of early and/or severe peritoneal dissemination. Furthermore, while the open Hartmann’s procedure is usually performed in such difficult and contaminated situations, the stoma closure rate after Hartmann’s procedure is known to be low (20–30%) [3, 4], implying that the stoma often becomes permanent. These poor oncological and functional outcomes are serious concerns.

From this perspective, surgery after controlling inflammation may be a more appropriate approach than immediate surgery in these patients. The absence of inflammation makes this situation easier to control. Although complete removal of cancer cells at the microscopic level in previous abscess areas may sometimes be realistically difficult, the completeness of resection can be much better than that achieved in immediate uncontrollable surgery under severe inflammation. Furthermore, anastomosis without a permanent stoma should be positively considered, as in our case, and laparoscopic or robotic surgery should also be adopted in skilled institutions. These functional merits have become increasingly important in the current era of preserved function and minimally invasive surgeries, and are also relevant for oncological outcomes.

Several options are available for achieving better control of inflammation. Bowel rest and antibiotics have apodeictic benefits. Stoma construction to divert the inflamed area is also beneficial, although the decision to perform this should be made by taking into account the patient’s quality of life even when the stoma will be temporary and have a favorable course, as in our case. Percutaneous drainage is the standard treatment for intra-abdominal abscesses. In colonic diverticulitis, percutaneous drainage in addition to bowel rest and antibiotics is recommended for an abscess more than 3–5 cm in diameter [5, 6], but the indications for malignancies should be carefully considered with the risk of fistula recurrence. Although fistula recurrence is thought to be rare, and similar treatments for other malignancies are widely accepted if necessary (e.g., fine needle aspiration for pancreatic cancer and percutaneous transhepatic biliary drainage for bile duct cancer) [7, 8], careful discussion about the indications is essential. The patient in the present study showed an extremely large intra-abdominal abscess approximately 13 cm in diameter, which could lead to sepsis, and percutaneous drainage was necessary. We are certain that our management strategy that included emergent percutaneous drainage was more appropriate than emergent surgery in this frail patient even after considering the risk of fistula recurrence.

The second important aspect of this case was the management of LACC. NAC has recently been considered a promising novel strategy to overcome the poor prognosis of LACC. NAC has been reported to yield a higher complete resection rate and even improved overall survival in comparison with conventional strategies (i.e., curative surgery first followed by ACT), and is described as a selectable option for T4b LACC by the latest guidelines from the National Comprehensive Cancer Network (NCCN) [9, 10]. However, the optimal NAC regimen remains unclear. The NCCN guideline describes 5-fluorouracil plus oxaliplatin (FOLFOX) or capecitabine plus oxaliplatin (CapeOX). Previous clinical trials also chose FOLFOX and failed to reveal the additional benefits of anti-epidermal growth factor receptor (EGFR) agents [9, 11].

The tumor in our case was marginally resectable, indicating that the situation was not completely the same as that associated with NAC, and the patient was too frail to undergo combination therapy with cytotoxic agents; therefore, we decided to perform preoperative Pmab monotherapy for the unresectable colorectal cancer in this frail patient. Anti-EGFR agents are well known for their ability to induce early tumor shrinkage, and anti-EGFR agent monotherapy is reported to be feasible even for frail patients [12, 13]. Therefore, Pmab monotherapy was thought to be appropriate as a curative resection-directed preoperative chemotherapy for this frail patient with a bulky tumor. A previous case report also described the effectiveness of preoperative Pmab use in a similar case of marginally resectable sigmoid colon cancer [14]. The optimal duration of NAC for LACC still remains unclear and should be evaluated on a case-by-case basis. In our patient, after seven cycles of Pmab monotherapy for approximately 3 months, we concluded that curative resection was appropriate, considering both the oncological perspective and the patient's general ability to endure the operative invasion. The guidelines based on clinical trials are generally intended for robust patients in their prime. In fact, almost all participants in previous clinical trials of NAC with FOLFOX-based regimens for LACC were under 75 years, even though the trial protocols did not prescribe upper age limits, suggesting that physicians realistically assessed elderly patients, such as the patient in our case, as not appropriate for the protocol regimens [9, 11]. Although evidence-based medicine is important, tailor-made chemotherapy based on the patient’s frailty is also necessary in the current era of an aging society. Thus, while preoperative anti-EGFR agent use for LACC has little evidence to date, our choice may have been one of the keys to the favorable course in this patient. The indication for ACT in our patient, the final pathological stage IIA, presents another interesting aspect of this case. In general, the possibility of down-staging when judging the indication of ACT for preoperative treatment cases in colorectal cancer should be considered, although down-staging may not be likely in our patient considering the minimal pathological tumor regression. Furthermore, perforation is generally associated with poor prognosis in colorectal cancer [15, 16], even in cases of stage II colon cancer, and ACT should be considered based on patient preference according to the NCCN guidelines. Given these factors and our patient’s preference, oral fluoropyrimidine-based ACT was selected.

## Conclusions

This study reports a successful case of curative resection after percutaneous drainage followed by preoperative Pmab monotherapy for locally advanced sigmoid colon cancer with a huge intra-abdominal abscess in an elderly and frail patient. The patient was recurrence-free and showed no significant organ dysfunction. The management of intra-abdominal abscesses and the tailor-made preoperative chemotherapy, which took into account the patient’s frailty, may be the key factors leading to this favorable course. Our experience is valuable for managing similar cases in the current era of an aging society.

## Data Availability

Not applicable.

## Abbreviations

LACC: Locally advanced colon cancer
ACT: Adjuvant chemotherapy
NAC: Neoadjuvant chemotherapy
Pmab: Panitumumab
CT: Computed tomography
NCCN: National Comprehensive Cancer Network
EGFR: Epidermal growth factor receptor
